# Screening mammography: sparing the emperor's blushes

**DOI:** 10.1002/cam4.859

**Published:** 2016-09-28

**Authors:** Saroj Niraula

**Affiliations:** ^1^ Medical Oncology and HematologyUniversity of Manitoba and CancerCare Manitoba675 McDermot AvenueWinnipegManitobaCanadaR3E 0V9

**Keywords:** Breast cancer, mammography, overdiagnosis, overtreatment, screening

## Abstract

Differing interpretations about evidence on benefits and harms of screening mammography has led to conflicting recommendations among different jurisdictions that range from intensive screening starting at age 40 to no screening at all. Despite broad attention of scientific and nonscientific media, evidence suggests substantial discrepancy between real and perceived benefits of screening mammography among women. In this commentary, underlying concept of mammographic screening, limitations in primary evidence, results from secondary evidence, and existing misunderstandings are underscored with a critical gaze at available information.

Most government programs are initiated based on positive theoretical utility, and usually with good intentions. However, once established, such programs become luxury of certain group that has immense interest to preserve them regardless of their eventual practical utility—a reason why we find very few instances of a government program being revoked in the entire history. Screening mammography is one of the examples. Despite a common scientific evidence, recommendations about screening mammography vary among jurisdictions ‐ American Cancer Society updated recently their guidelines revising recommended age of initiation of screening mammogram from 40 to 45 years; Canadian task force on preventive health care recommends it for women starting at age 50; whereas, the Swiss medical board recommended recently against any new mammographic screening programs 1, 2.

Localized breast cancer in itself is never fatal. Therefore, the sole purpose of screening mammography is to detect breast cancer early, thereby preventing progression into an advanced form. An analysis from the Surveillance, Epidemiology, and End Results (SEER) database suggests no increase in the incidence of de‐novo metastatic breast cancer since 1975 3. Occurrence of stage III–IV breast cancer is similar between screened and nonscreened population—a study of >40,000 women with invasive breast cancer reports similar decline in advanced breast cancer in screened versus nonscreened populations 4. Report on a three‐decade experience of screening mammography suggests that despite >100% increase in the incidence of early‐stage breast cancer with screening, incidence of advanced breast cancer declined by a mere 8%‐ a phenomenon that can be explained easily by increased awareness. These finding together speak to the fact that screening mammography has not been able to prevent as was hoped for, the incidence of the fatal stage of breast cancer 5.

Concept of mammography relies on a traditional assumption that breast cancer grows in anatomic linearity—first involving the breast, then progressing to locoregional lymph nodes before involving the distant organs. However, breast cancer is an enormously heterogeneous group of diseases. Whereas some breast cancers are slow‐growing and may disseminate linearly, others will have (at least microscopically) involved distant organs before they are detected in the breast—identification of such cancers with screening mammogram is either not possible or is unhelpful.

Natural history of some screen‐detected breast cancers is likely different from nonscreen‐detected breast cancers. For example, an astonishing phenomenon was observed when upper age limit of screening mammography was updated in the UK from 64 to 69 years in 2001—there was a steep surge in incidence of new breast cancer, although these women were already being screened for many years while they were still under 64 years—such observation can only be explained if screen‐detected cancers regress with time 6. Another provocative study from Norway confirms such phenomenon 7.

A meta‐analysis of 10 clinical trials involving over 600,000 women by the Cochrane collaboration suggested only a modest reduction in breast cancer deaths attributable to screening, and that was counterbalanced by deaths from other causes without benefits in overall survival 8. Screening also increased the rate of mastectomies 8. Over half of the screened women receive at least one false alarm in form of “recall” which is frequently followed by a biopsy for a noncancer abnormality 9. In addition to physical inconvenience of biopsy, this invites considerable psychological and emotional distress difficult to justify in view of small relative benefits, if any. False‐positive screening mammograms are associated with significant psychological distress that persists even years after the mammograms are deemed normal 10. Furthermore, for every breast cancer related death prevented, 10 healthy women that would never be diagnosed with breast cancer receive the diagnosis and are subject to potentially debilitating treatments. Consequently, (over) treatment of overdiagnosed breast cancer leads to deaths from other causes that wipe out marginal effect on cause‐specific mortality. Possible higher mortality from breast cancer is suggested in screened women aged 40–49 compared to nonscreened 11.

And 90% of the clinical trials that evaluated screening mammography are old (conducted before the eighties) and are methodologically poor by modern standards as evidenced by the fact that the trials that report the largest benefits from screening mammography ironically used only one‐view mammography, screened the control groups only after 3–5 years, and screened at substantially prolonged intervals 8.

Survey of American women suggests that a vast majority of women (>70%) believed that screening mammography reduces the risk of death from breast cancer by at least half which is far from what the evidence suggests 2. In addition, women believed that 80 deaths from breast cancer would be prevented with the number of mammography that would actually only prevent one death. This underscores the exceptionally worrisome status of informed consent process in women's decision to undergo a screening mammography even in the most resourceful settings.

Timing of uptake of screening mammography around the world varies, but the death after diagnosis of breast cancer started to decrease consistently after introduction of tamoxifen and effective chemotherapies 12—the most reliable confirmation that modern treatments, not screening mammography has improved survival of women with breast cancer. If a medical intervention does not make people live longer, is associated with inconvenience, adds lasting distress, and can compromise quality of life, an affirmative utility on such intervention is impossible with any analysis.

If screening mammography is to be ever beneficial, that is possible only in carefully selected women based on individual assessment of their risks rather than of the population as a whole. Women with strong personal or family history and/or genetic conditions that increase the risk of breast cancer should be screened more vigilantly. Evidence‐based tools to estimate variations in harms and benefits have been published and may help inform women 9. On an individual level, the benefits and harms of any medical intervention are subjective, and trade‐off that a woman is willing to accept for a given benefit is best left at the discretion of the individual. Government programs should serve evidence‐based interest of consumers rather than that of those who survive off such programs. And, every woman has the right to up‐to‐date and unbiased information to make best judgment for herself before her next screening mammogram.

## Conflict of Interest

Author has no conflict of interest to disclose.

## References

[1] Canadian Task Force on Preventive Health C ; Tonelli, M. , S., Connor Gorber , et al. 2011 Recommendations on screening for breast cancer in average‐risk women aged 40–74 years. CMAJ 183:1991–2001. 22106103 10.1503/cmaj.110334 PMC3225421

[2] Biller‐Andorno, N. , and P. Juni . 2014 Abolishing mammography screening programs? A view from the Swiss Medical Board. N. Engl. J. Med. 370:1965–1967. 24738641 10.1056/NEJMp1401875

[3] Welch, H. G. , D. H. Gorski , and P. C. Albertsen . 2015 Trends in metastatic breast and prostate cancer‐lessons in cancer dynamics. N. Engl. J. Med. 373:1685–1687. 26510017 10.1056/NEJMp1510443

[4] Kalager, M. , H. O. Adami , M. Bretthauer , et al. 2012 Overdiagnosis of invasive breast cancer due to mammography screening: results from the Norwegian screening program. Ann. Intern. Med. 156:491–499. 22473436 10.7326/0003-4819-156-7-201204030-00005

[5] Bleyer, A. , and H. G. Welch . 2012 Effect of three decades of screening mammography on breast‐cancer incidence. N. Engl. J. Med. 367:1998–2005. 23171096 10.1056/NEJMoa1206809

[6] Jorgensen, K. J. , J. D. Keen , and P. C. Gotzsche . 2011 Is mammographic screening justifiable considering its substantial overdiagnosis rate and minor effect on mortality? Radiology 260:621–627. 21846758 10.1148/radiol.11110210

[7] Zahl, P. H. , J. Maehlen , and H. G. Welch . 2008 The natural history of invasive breast cancers detected by screening mammography. Arch. Intern. Med. 168:2311–2316. 19029493 10.1001/archinte.168.21.2311

[8] Gotzsche, P. C. , K. J., Jorgensen P. C., Gotzsche 2013 Screening for breast cancer with mammography. Cochrane Database Syst. Rev. 6:CD001877. 10.1002/14651858.CD001877.pub5 PMC6464778 23737396

[9] Welch, H. G. , and H. J. Passow . 2014 Quantifying the benefits and harms of screening mammography. JAMA Intern. Med. 174:448–454. 24380095 10.1001/jamainternmed.2013.13635

[10] Brodersen, J. , and V. D. Siersma . 2013 Long‐term psychosocial consequences of false‐positive screening mammography. Ann. Fam. Med. 11:106–115. 23508596 10.1370/afm.1466 PMC3601385

[11] Narod, S. A. , P. Sun , C. Wall , et al. 2014 Impact of screening mammography on mortality from breast cancer before age 60 in women 40 to 49 years of age. Curr. Oncol. 21:217–221. 25302030 10.3747/co.21.2067 PMC4189562

[12] Early Breast Cancer Trialists' Collaborative Group . 1988 Effects of adjuvant tamoxifen and of cytotoxic therapy on mortality in early breast cancer. An overview of 61 randomized trials among 28,896 women. N. Engl. J. Med. 319:1681–1692. 3205265 10.1056/NEJM198812293192601

